# The twenty most charismatic species

**DOI:** 10.1371/journal.pone.0199149

**Published:** 2018-07-09

**Authors:** Céline Albert, Gloria M. Luque, Franck Courchamp

**Affiliations:** Ecologie Systématique Evolution, Univ Paris-Sud, CNRS, AgroParisTech, Université Paris-Saclay, Orsay, France; Smithsonian Conservation Biology Institute, UNITED STATES

## Abstract

Charisma is a term commonly used in conservation biology to describe species. However, as the term “charismatic species” has never been properly defined, it needs to be better characterized to fully meet its potential in conservation biology. To provide a more complete depiction, we collected information from four different sources to define the species currently considered to be the most charismatic and to understand what they represent to the Western public. First, we asked respondents of two separate surveys to identify the 10 animal species that they considered to be the most charismatic and associate them with one to six traits: *Rare*, *Endangered*, *Beautiful*, *Cute*, *Impressive*, and *Dangerous*. We then identified the wild animals featured on the website homepages of the zoos situated in the world’s 100 largest cities as well as on the film posters of all Disney and Pixar films, assuming in both cases that the most charismatic species were generally chosen to attract viewers. By combining the four approaches, we set up a ranked list of the 20 most charismatic animals. The majority are large exotic, terrestrial mammals. These species were deemed charismatic, mainly because they were regarded as beautiful, impressive, or endangered, although no particular trait was discriminated, and species were heterogeneously associated with most of the traits. The main social characteristics of respondents did not have a significant effect on their choices. These results provide a concrete list of the most charismatic species and offer insights into the Western public’s perception of charismatic species, both of which could be helpful to target new species for conservation campaigns.

## Introduction

Conservation programmes for endangered species work better when supported by the target public (as well as NGOs and governments) in terms of fundraising, policymaking, or participatory programmes. As a result, efficient communication campaigns from conservationists are of paramount importance [1]. Due to the tremendous number of species of conservation concern, it has become common practice to focus on particular species as surrogates for conservation studies and programmes, whether for research or communication purposes [1].

**Fig 1:**
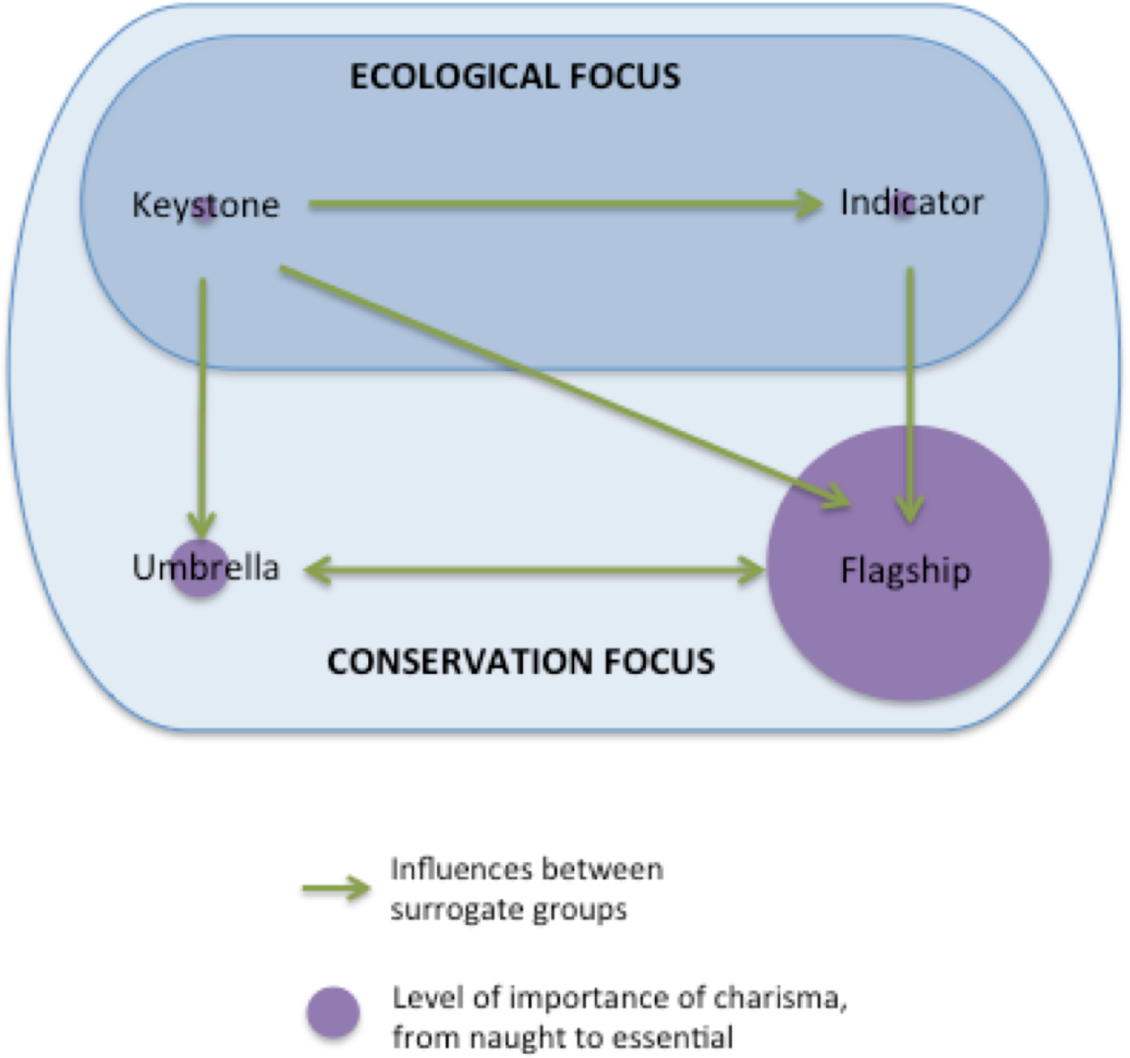
Relationships between charisma and the four main surrogates for conservation studies.

The four most prominent surrogate species types are indicator, keystone, umbrella, and flagship species (Fig 1; see [2] for another classification). The former two represent species of ecological relevance: indicator species are commonly chosen, because they quickly respond to minimal changes in the environment or biodiversity loss, while keystone species play important ecological roles in the integrity of the ecosystem structure and functioning [3,4]. Umbrella and flagship species are mainly used as tools for conservation. The major purpose of umbrella species is to protect biodiversity. Indeed, these species usually have a large home range, so their protection is beneficial for species sharing their habitat [5]. Lastly, flagship species are used to increase public awareness about conservation issues and/or promote fundraising [6–8] by focusing conservation and communication activities on species that people feel concerned about. Similar to umbrella species, flagship species are usually large-sized animals, often selected based on their level of endangerment [9]. In addition, it has been proposed that flagship species are non-biological surrogate groups that focus on species traits instead of the species itself [10], and/or species with traits that assemble relatively coherent networks of associations in pre-existing cultural frames and the political economy [8]. It should be noted that some species can fulfil several proxy roles; for example, the tiger (*Panthera tigris*) is both an umbrella species [11,12] and a flagship species [13,14]. This led to the establishment of the combined concept of “flagship umbrellas” [1].

Charisma is another characteristic commonly used to describe flagship species [6,15]. It is therefore not a type of surrogate species, but rather a relational trait (or suite of traits) of a species. In conservation, the role of charisma may be suggested in public communication [10,16] and the funding process [6,17]. As people are naturally attracted to charismatic species, they are more willingly fund conservation programmes focusing on such species [15,18,19]. However, the use of charismatic species to elicit funding is debated [3,6,20], because this may cause conservationists to focus their protection programmes and studies on areas that are not the most urgent in ecological terms [21,22]. Consequently, the public may underappreciate biodiversity or overlook non-charismatic species in need of conservation attention [23].

As a word of ecclesiastic origin, charisma is more commonly used to describe people. According to the Oxford Dictionary, it describes “the powerful personal quality that some people have to attract and impress other people” without any references to other organisms such as plant and animal species, even though it is increasingly used to describe nonhuman species. According to the Web of Science database, it was first used in 1990 by Noss [24] who mentioned it as one of the required attributes of flagship species. Since then, studies using this term have increased exponentially, culminating in more than 300 publications to date (Web of Science; https://webofknowledge.com). The term is used both as a characteristic to describe flagship species [15,25] and as a surrogate species [26,27]. Some studies have attempted to characterize this term. Indeed, the concept of non-charisma was discussed by Lorimer (2007) [6] who divided nonhuman charisma into three groups: ecological (ethological perspective on the human/environment perception), aesthetic (referring to species behaviour or appearance, thus dealing with human emotions), and corporeal (referring to “affection and emotions engendered by different organisms in their practical interactions with humans over varying time periods”). In addition, Jepson et al. [28] theorized nonhuman agency, explaining how charismatic animals could be co-actors of their own conservation, since they have the capacity, whether intentionally or not, to affect conservation outcomes. However, this concept has never been fully and unambiguously defined in the literature [6].

To better understand the use and usefulness of charismatic species and advance the debate, it appears important to fully define this term, both in terms of semantics and thus defined species. On a practical level, it is a subjective concept, and its content is therefore associated with the target public, which needs to be clearly identified. Here, we focus on Western countries from which most conservation funding comes. As a first step towards providing such a definition, it is important to know which species the public considers to be charismatic and which traits are associated with this charisma. Other studies have focused on charisma in nonhuman species [17,29], but they involved a closed choice (selection among a predetermined collection) and were conservation-oriented, which restricted the breadth of possibilities in the results. Therefore, we used a rigorous, open, and non-restrictive approach in order to ensure that the definition was as least oriented as possible. In this manner, we used data from four complementary sources to illustrate the charisma of species for the Western public, which enabled us to compile a list of 20 animals considered to be the most charismatic. These sources were the following: (i) a large-scale online survey, (ii) a questionnaire given to primary school children in Western Europe, (iii) the animals displayed on the websites of zoos situated in the 100 largest cities in the world, and (iv) the animals featured on the film posters of animated movies produced by Disney and Pixar. All were open (no pre-suggested species) and not conservation-oriented. Two sources involved direct questions to the public, while we worked under the assumption that the species displayed on the zoo websites and film posters would be selected by communication experts based on their general appeal to the public. Collectively, these data were considered to be representative of animals regarded to be the most charismatic.

## Materials and methods

### Methods

We focused on the public in Western societies, as they are often a major source of conservation funding. To establish a list covering the opinions of a broader public, we used four different sources ranging from surveys to proxies. The use of four complementary sources also lessened the possible bias that could be associated with one particular method. In addition, we asked survey respondents to link each species with six traits. We made the assumption that the traits associated with the species in the survey were the same for the two other lists. To support this decision, we tested the correlation between the ranks of the four lists (see Statistical analyses below). We then combined the four lists into a single one, while giving them equal weight.

#### Online survey

An internet survey was conducted through social networks (S1 Text) over a period of three months (May to July 2011). Using a specifically designed website, we developed a survey that aimed to determine which species people considered to be the most charismatic and which traits (six choices) they associated with these species. The survey comprised two webpages. The first section recorded the characteristics of the respondents (age, sex, education level, language, and country), and their choice of 10 species considered to be the most charismatic, in no particular order. It was specified that they should only consider wild animal species. In the second section, respondents were asked to associate one or several of the following characteristics with the 10 selected species: *Beautiful*, *Dangerous*, *Impressive*, *Cute*, *Rare*, and *Endangered*. These characteristics appeared in a random order for each respondent. They were selected empirically so as to cover most of the feature categories found to be potentially associated with charisma in the literature. Although self-explanatory, they were purposely left undefined so as to represent the subjective appreciation of each participant. Among the six proposed traits, two described appearance (*Beautiful* and *Cute*), two defined the relationship with humans (*Dangerous* and *Impressive*), and two related to conservation (*Endangered* and *Rare*). The survey (http://max2.ese.u-psud.fr/epc/conservation/pages/Franck/Charismanimals/Index.html) was available in four languages (English, French, Spanish, and Italian), and an incentive (i.e., a slideshow of funny photos) was offered as a reward at the end of the survey to encourage respondents to finish the survey and distribute it further. We collected answers from a total of 4,522 respondents from 69 countries, with the majority from Western Europe, the USA, Australia, and New Zealand. As there were very few responses in the Italian language, we removed them from the analyses. These data were collected anonymously.

#### School surveys

The same survey was conducted with children aged around 10 years in three primary schools located in three different countries: England (London), Spain (Córdoba), and France (Limours, near Paris). The schoolteachers first explained the meaning of the term “charismatic” as attractive, appealing, and preferred, while taking care not to mention the six traits or their synonyms. The explanation was purposely kept concise, and pupils were given 10–15 minutes to complete a paper questionnaire with the same questions as found in the web survey. We collected 224 usable complete questionnaires.

#### Zoo webpages

We collected the names of wild animal species displayed on the homepage of the official websites of major zoos from the 100 largest cities in the world (https://en.wikipedia.org/wiki/World's_largest_cities accessed on 16-04-2012). When several species were displayed, all were recorded. Any unidentifiable species (i.e., unclear photos, cartoon depictions) were discarded.

#### Animated film posters

We recorded the names of wild animal species featured on the American version of the film posters of all animated movies produced by Disney (https://en.wikipedia.org/wiki/List_of_Disney_theatrical_animated_features accessed on 29-03-2012) and Pixar (https://en.wikipedia.org/wiki/List_of_Pixar_films accessed on 29-03-2012), when recognizable. We discarded any imaginary (e.g., dragons) or extinct (e.g., dinosaurs) species.

### Taxonomic issues

Whether in the surveys, zoo websites, or film posters, the animals were not always given or identifiable at the species level. Given the purpose of our study, we did not systematically aim to assess the precise taxonomic entity, as in most cases the public would not make distinctions in terms of charisma. This explains why we retained responses such as “elephant”, despite the fact that it can represent two species from two genera and two continents. Similarly, many of the animals nominated in our survey corresponded to different species (e.g., gorillas, zebras, crocodiles) or subspecies (e.g., giraffes, tigers). Nevertheless, we discarded nominee animals that belonged to an excessively broad taxonomic group such as “fish” and “bird” and could not be situated in a more precise group. For the other imprecise denominations, the nominated animals were refined, because they corresponded to visually different species from very charismatic groups such as bears, sharks, eagles, dolphins, whales, snakes, and apes. As a result, we designed a second online survey (http://max2.ese.u-psud.fr/epc/conservation/pages/Franck/Charisma2/) where respondents–not necessarily the same as in the first survey–could state whether they had a specific species in mind when mentioning each of these groups. If the answer was affirmative, they were asked to select a specific species among the six most common species in that group. The six species were randomly presented on a separate page with very similar photos or pictures. The information obtained in the second survey provided a correcting factor to rank these species in the first survey. For example, the first survey yielded 1,192 responses that listed “eagle”, but the second survey indicated that the golden eagle (*Aquila chrysaetos*) was intended in 39.3% of cases and the bald eagle (*Haliaeetus leucocephalus*) in 26.4% of cases. Consequently, the 1,192 responses with the mention of “eagle” were subdivided, with 468 responses being added to the initial 145 responses that specifically mentioned golden eagles, and 315 to the initial 18 mentions of bald eagles, thus providing a final ranking for these species.

### Statistical analyses

Except for the ranking between the film posters and children’s survey, all other rankings were correlated with each other (S1 Table). We combined all scores (i.e., number of survey votes and number of mentions for zoos and film posters) for each species into an overall score, thus giving us the final rank of the most charismatic species.

**Fig 2:**
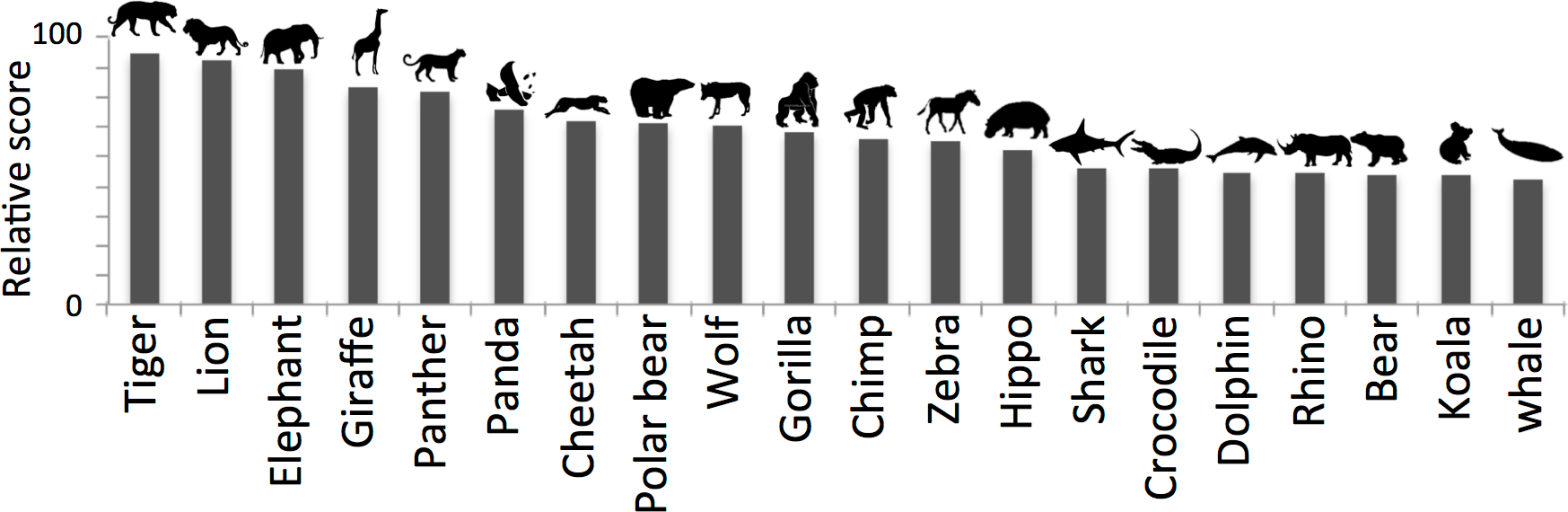
Twenty most charismatic animal species, in ranking order. The relative score, i.e., the score for the four survey sources relative to the first ranked (tiger), shows a relatively homogeneous distribution.

All of the following analyses were conducted using the online survey results alone. We used the chi-square test of homogeneity to assess whether the sociocultural categories (age, education level, language, and gender) were homogeneous. First, we removed from the dataset the responses with missing sociocultural information, thus resulting in a total number of 4,520 respondents. To assess any redundancy between the three main pairs of traits (“*Rare/Endangered*”, “*Beautiful/Cute*”, “*Dangerous/Impressive*”), we used McNemar’s test to assess any correlations among them. As none of the pairs were correlated (S2 Table), we used all six traits for the analyses. To assess which species contributed the most to trait proportions (Fig 2), we used the chi-square test of homogeneity to test for any differences between the traits of each species and global trait selection.

To assess the potential associations of each trait with the 20 most charismatic species, we performed six generalized linear mixed models (GLMM) in which the dependent variable was one of the six traits and the main independent variable was the species with 20 categories. As the response for each trait was either selected or non-selected by the questionnaire respondents, a binomial distribution of errors was used with a *logit* link function. As the data from people of similar age, gender, education level, or language were correlated, we included four random factors corresponding to these variables in each model: age (four categories), gender (two categories), education level (five categories), and language (three categories).

We assessed whether the 20 most charismatic species were associated with any sociocultural category. Sociocultural categories (age, education level, language, and gender) were nominal data with 2 to 5 levels (study level = 5, age = 4, language = 3, gender = 2). We conducted multiple correspondence analyses (MCA) with a data matrix formed by the frequency of each animal for each level of a given sociocultural category. In addition, we examined the association between species traits and sociocultural categories using MCA, which included the frequency of species traits for each sociocultural category.

All statistical analyses were conducted with R software [30], using “lme4” [31] and “ade4” packages [32].

## Results

The 20 most charismatic animals and their overall ranking are shown in Fig 2. The majority of species are large-sized mammals (four big cats, three bears, one canid, two primates, two cetaceansn and five large ungulates), while the remaining three are a smaller mammal (koala, *Phascolarctos cinereus*), a large reptile (crocodile, *Crocodylus sp*.), and a large Chondrichthyan (great white shark, *Carcharodon carcharias*). Ten are strictly predators, while seven are herbivores; all are long-lived species. Seventeen are terrestrial species and three marine species. Their relative scores are similar and do not show any species to be markedly more charismatic than another.

**Fig 3:**
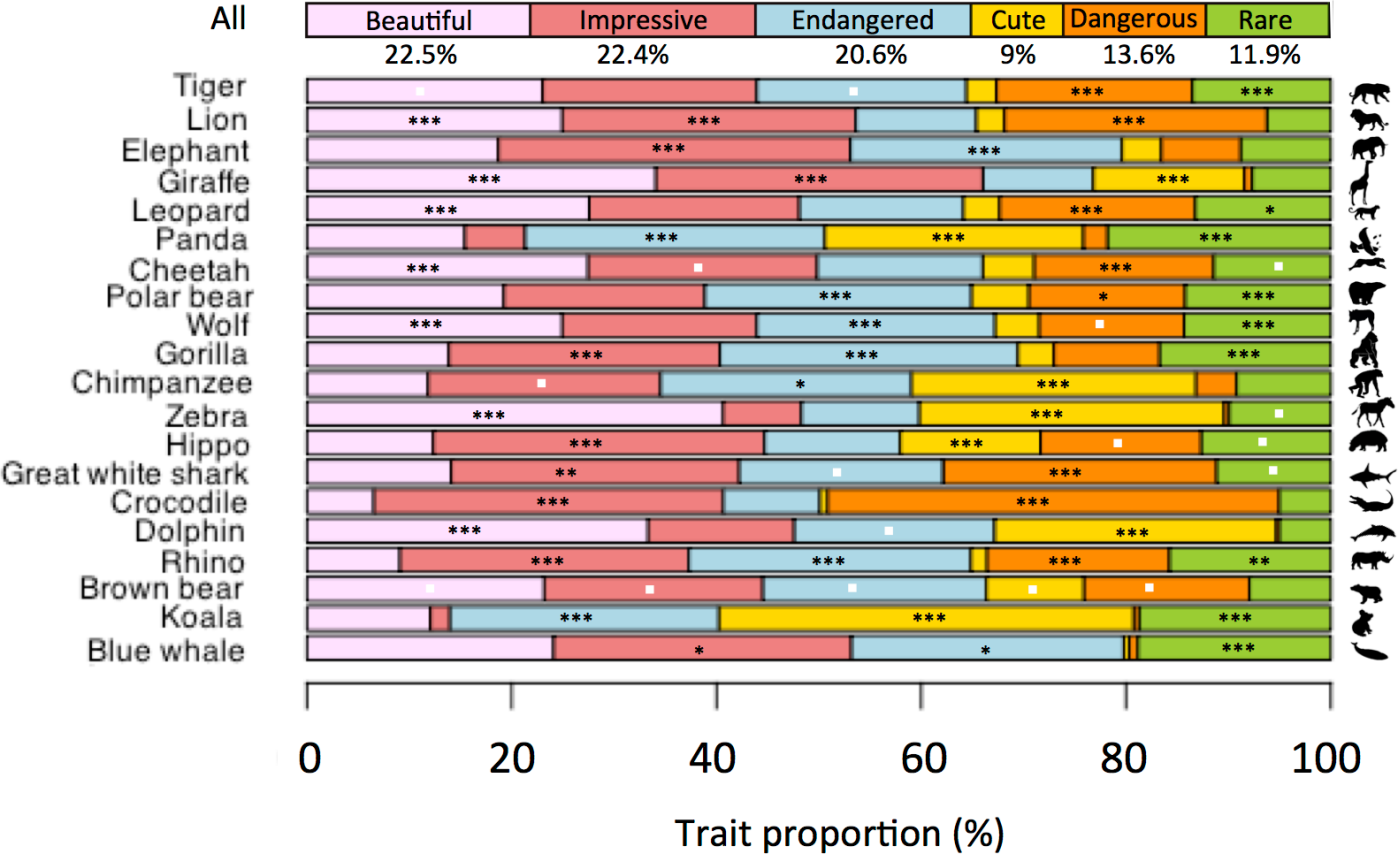
Proportions of traits for the 20 most charismatic species compared to the global proportion. The chi-squared test, used to assess whether trait proportions are significantly more attributed to a given species, is represented by the p-value significance *** <0.0001, ** <0.01, * <0.05 and by a white square for non-significance. The p-value is not represented when a trait proportion is less often attributed to a species (see details in S3 Table).

Regarding the species traits selected by respondents, none was over- or underrepresented (Fig 3 and S3 Table), with proportions ranging from 9% to 22.5%. The McNemar test, used to assess the associations between traits, revealed independence between all, except for the traits *Beautiful* and *Impressive* (S1 Table).

**Fig 4:**
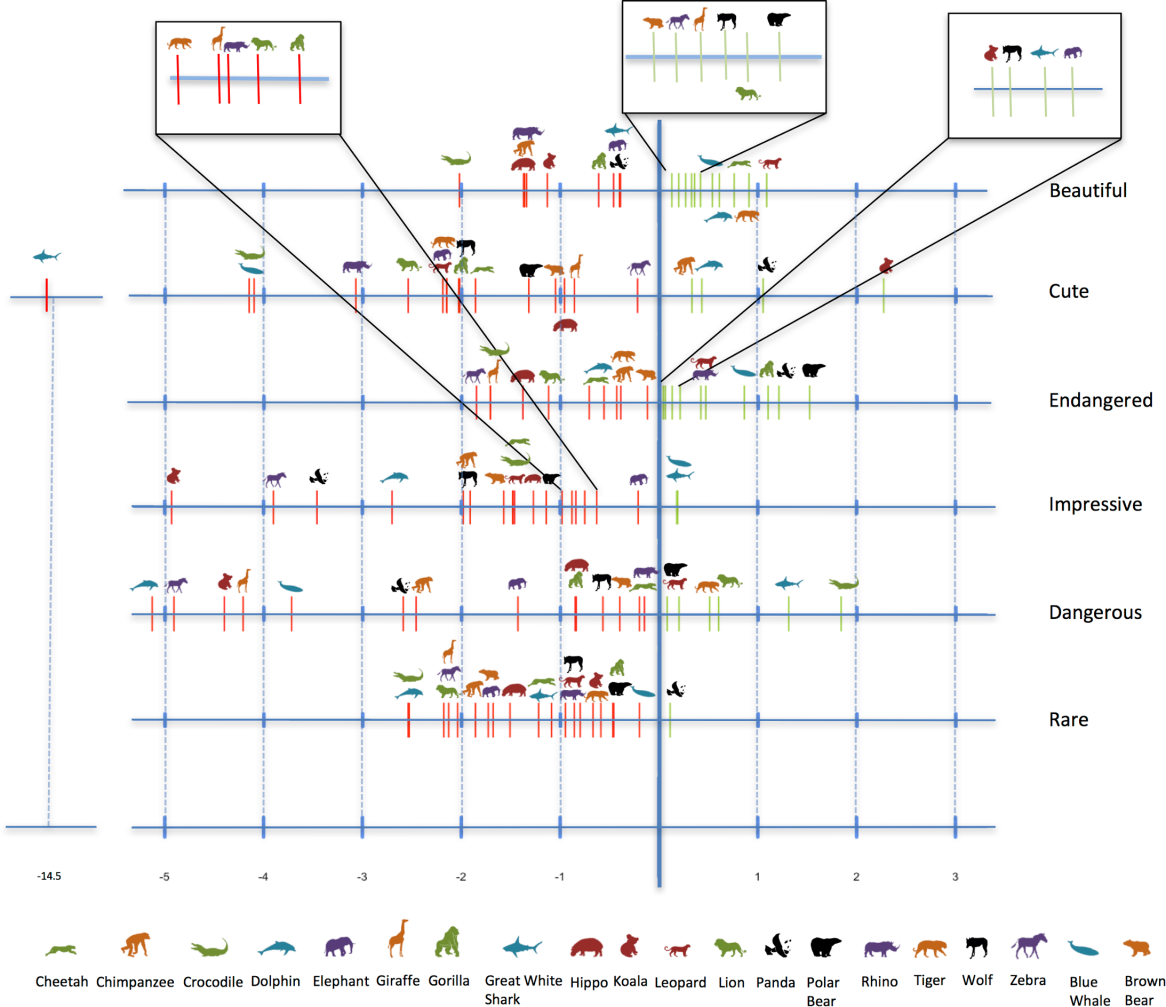
Illustration of the GLMM estimates, which represent the association between species and traits. See S4 Table for the GLMM estimates.

All species were described by all six traits (except one: *Cute* was never selected for the great white shark). Differences in the frequencies of traits for each species generated distinct trait profiles, but the associations between traits and species did not show any strong pattern. When assessing whether some traits were more frequently associated with certain species, GLMM showed that each species trait had a different pattern of species association. These traits more often discriminated negatively (Fig 4 and S4 Table), because each respondent generally selected only a few traits, with most traits not being selected in each questionnaire. A few species were discriminated by both negative and positive associations (e.g., the koala is *Cute* but not *Impressive*, while the crocodile is *Dangerous* but not *Beautiful*), while other species were more characterised by the presence of a given trait (e.g., whales, gorillas, pandas, and polar bears are *Endangered*) or its absence (e.g., dolphins and zebras are not *Dangerous*).

There was an uneven distribution of respondents with regard to gender, age, and language (gender: with 69.9% of female responents, χ^2^(1) = 710.14, p<0.001; age: 53.7% of respondents aged between 26 and 55 years, χ^2^(3) = 2843.1, p<0.001; and language: French: 86.7%, English: 9.00%, and Spanish: 4.3%, χ^2^(3) = 5797.4, p<0.001). However, this did not affect the analyses. According to our results, the sociocultural categories did not drive the choice of species or species traits. Both the first MCA (S5 Table), confronting sociocultural categories and species, and the second MCA (S6 Table), confronting sociocultural categories and species traits, revealed the absence of a clear pattern.

## Discussion

Thanks to the open, non-conservation-oriented approach of this study, we were able to rank the 20 most charismatic species according to the views of the general public in Western countries, as identified from both direct and indirect sources. These species were ranked in the following order: tiger, lion, elephant, giraffe, leopard, panda, cheetah, polar bear, wolf, gorilla, chimpanzee, zebra, hippopotamus, great white shark, crocodile, dolphin, rhinoceros, brown bear, koala, and blue whale. The 20 most charismatic species are overrepresented by comparably larger species (19/20), mammals (18/20), and terrestrial species (17/20). Most (11/20) are African species, with nine from savannah ecosystems. The overrepresentation of mammals was expected given their overrepresentation in conservation biology and communication campaigns [33–37] as well as in the scientific literature [33,38], not to mention the general appeal of species that are phylogenetically or physiognomically closer to humans [29,39,40]. Ward et al. [41] also found that zoo exhibits of larger animals are preferred by both adults and children. Although they did not predominate, the presence of the great white shark and crocodile suggests that non-mammals can also be regarded as charismatic by the general public.

It is noteworthy that many of the 20 species are or have recently been the focus of conservation campaigns. For example, most of the species listed as flagship by Clucas et al. [9] are also found among the 20 most charismatic species identified in this study. As already proposed [42], it is quite possible that the perception of charisma has been partly built through these campaigns (e.g., panda and polar bear). Yet it is impossible to determine how much of their charisma comes from the effects of conservation campaigns, and how much of their prior charismatic status made these species [43,35]appropriate choices to be conservation programme icons.

Species traits were homogeneously distributed, and all 20 species were described by most traits, with different patterns of associations. Nevertheless, *Beautiful*, *Impressive*, and *Endangered* were more often selected. The traits were predefined, and no additional traits could be submitted. Although we believe that these traits encompass the essence of charisma, this necessarily limited our definition of charismatic species. Notably, the choice of these six traits was mostly based on the conservation science literature that uses the term “charismatic species”. We might, however, have found other relevant categories within the theoretical literature on nonhuman charisma and agency or in the wider literature on charisma in the social and management sciences. This is indeed a limitation of this study, not in the sense that the resulting list would have varied, but rather that the results might have been exploited differently if more or better categories had been identified.

Within our sample, the sociocultural category did not drive the choice of any species in particular. It appears that having a large body size and being a mammal are the primary features that make a species charismatic, and that other characteristics are only secondary, without a clear profile emerging. A species can be charismatic, because it is endearing or terrifying. Therefore, one of the most striking results of this study is that the 20 charismatic species cannot be described by one particular profile.

The six proposed traits pertained to three larger characteristics: appearance (*Beautiful* and *Cute*), relationship with humans (*Dangerous* and *Impressive*), and conservation status (*Endangered* and *Rare*). Each pair was however non-redundant, as shown by the lack of association of trait pairs. It is interesting that not all species are deemed charismatic because of their visual appeal (*Beautiful* or *Cute*). Some are visibly selected only because they are frightening (e.g., great white shark and the crocodile). It is also noteworthy that only one species is consistently described as *Rare* (panda), and only half of the species are described as *Endangered*, despite the fact that they are all endangered [44]. Interestingly, the panda is the only species associated with both traits, suggesting that in the public’s view, an endangered species is not necessarily rare. Hence, as many traits or associations of traits emerge from our results, charismatic species cannot be associated with a particular trait profile.

Survey respondents were French, English, and Spanish speakers, mostly from Western countries. Therefore, they are not representative of the global human population. However, since charisma is a characteristic used mostly for conservation purposes (leaving aside commercial considerations here), the focus of our sampling reflected the countries of origin where the public most actively funds conservation programmes. Regarding the sociocultural category of respondents, species choice, and trait selection, our results do not highlight any pattern of selection, suggesting that conservation campaigns should not be overly constrained regarding these characteristics.

To this Western public, most of the listed species are exotic. Wolf and brown bear are the obvious exceptions, but given their reintroduction into most parts of Western Europe after decades of absence, it is possible that they are also perceived to be exotic species. They are also large predators, which is a recurrent feature in the list, with dangerousness seemingly exerting an appeal for many respondents. In addition, respondents of both surveys (internet users and school children) proposed a large number of species at different taxonomic levels. As we were interested in the species level, we adjusted answers using a second internet survey and excluded higher taxonomic groups (see Methods). Yet it is noteworthy that an accurate species name is of less importance to the public than to scientists. These two points suggest either a poor knowledge or poor interest in local species [27,45] in addition to a limited knowledge of species names.

Consequently, according to our results, we can only propose a rather flexible description of charismatic species for the Western public as being a characteristic describing species as preferentially–but not necessarily–a large, terrestrial, and exotic mammal. This is in accordance with recent studies (e.g., [46]). Therefore, charismatic species are close to iconic species, but it is important to distinguish between them, as iconic species refer to culturally important species that people venerate (i.e., glorify, deify, offer respect), which is not the case with charismatic species. The charisma of species is of special importance in conservation marketing campaigns, and thus the flexibility of these criteria is an encouraging result, as it greatly broadens the spectrum of species that can be used in this context. Charismatic species are often easily anthropomorphized, being presented with forward-facing eyes for example [40], but this not always the case, as many other species are routinely anthropomorphized in many media. As charismatic species do not necessarily need to be visually appealing and can even be frightening, this results in a kaleidoscope of possibilities. This could give us the opportunity to target other endangered species that respond to the main traits highlighted here, such as the okapi (*Okapia johnstoni)*, muskox (*Ovibos moschatus*), tapir (*Tapirus sp*.), and plains bison (*Bison bison*). However, the fact that most of the identified species are large terrestrial mammals does not mean that aquatic, non-mammal, or smaller species could not be used successfully, depending on both the target public and campaign objectives. Finally, the flexibility of the charisma criteria should be seen as an asset not only for conservation campaigns, but also more broadly for a better exploitation of umbrella and flagship species in conservation.

## Supporting information

S1 Text Dissemination of the online survey. (DOCX)

S1 Table Correlations among the ranking lists of charismatic species coming from the four different sources. We show Spearman’s rank coefficients for each correlation pair and in brackets p-values.
(DOCX)

S2 Table p-value of the McNemar test. The only association is a negative association between *Impressive* and *Beautiful*.
(DOCX)

S3 Table p-value of the Chi-square. Test of the difference between mean proportions of traits and proportions of species traits. See Fig 2.
(DOCX)

S4 Table Estimates of the GLMM. Illustration in Fig 4.
(DOCX)

S5 Table MCA1 (Species VS respondents characteristics). (DOCX)

S6 Table MCA2 (Species traits VS respondents traits). (DOCX)
