# Supplementary material for: The twenty most charismatic species
Source: PLoS One. 2018 Jul 9;13(7):e0199149. doi: 10.1371/journal.pone.0199149 (PMC6037359; doi:10.1371/journal.pone.0199149)
Supplement: S1 Text — (DOCX) [file pone.0199149.s001.docx]

S1 Text: Dissemination of the online survey.

This online survey was conducted from May to July 2011. The first week of the survey, the link of was shared through discussion forums, as well as social, personal and professional networks. It was also proposed at the visitor of a scientific museum.

A link to the survey was shared on discussion forums on Yahoo ([https://groups.yahoo.com/)](https://groups.yahoo.com/%29). We carefully selected groups to not be species specific (i.e. avoiding forums focused on cats, hunting, marine life and so on). Then the ten first forums of the following categories received the invitation to answer the survey ([Hobbies & Crafts](https://groups.yahoo.com/neo/dir/1600062280)/Hobbies, excepted Aquarium et Taxidermy; [All](https://groups.yahoo.com/neo/dir)/[Entertainment & Arts](https://groups.yahoo.com/neo/dir/1600016068)/[Comics and Animation](https://groups.yahoo.com/neo/dir/1600017755)/ ; [Hobbies & Crafts](https://groups.yahoo.com/neo/dir/1600062280)/Models, excepted Horses and Science Fiction ; [All](https://groups.yahoo.com/neo/dir)/Recreation & Sports, excepted Others). 
In addition to these discussion forums, the survey was sent to the scientific department of the authors (at the University of Orsay), which was at the time about 130 people. These collaborators were asked to forward the invitation to their own professional and personal networks (e.g., through Facebook accounts).

Finally, the survey was proposed during the two months at the “Palais de la Découverte” in Paris, France, were an intern proposed visitors to answer the online survey through an iPad.
