# Supplementary material for: The twenty most charismatic species
Source: PLoS One. 2018 Jul 9;13(7):e0199149. doi: 10.1371/journal.pone.0199149 (PMC6037359; doi:10.1371/journal.pone.0199149)
Supplement: S1 Table — We show Spearman’s rank coefficients for each correlation pair and in brackets p-values. (DOCX) [file pone.0199149.s002.docx]

S1 Table: Correlations among the ranking lists of charismatic species coming from the four different sources. We show Spearman’s rank coefficients for each correlation pair and in brackets p-values.

|  | **Children** | **Web Survey** | **Movies** | **Zoo websites** |
| --- | --- | --- | --- | --- |
| **Children** | - | - | - | - |
| **Web Survey** | 0.75[0.0003] | - | - | - |
| **Movies** | 0.36[0.169] | 0.69[0.003] | - | - |
| **Zoo websites** | 0.59[0.03] | 0.83[<0.0001] | 0.64 [0.028] | - |
