# Supplementary material for: The twenty most charismatic species
Source: PLoS One. 2018 Jul 9;13(7):e0199149. doi: 10.1371/journal.pone.0199149 (PMC6037359; doi:10.1371/journal.pone.0199149)
Supplement: S2 Table — The only association is a negative association between Impressive and Beautiful. (DOCX) [file pone.0199149.s003.docx]

**S2 Table:** p-value of the McNemar test. The only association is a negative association between *Impressive* and *Beautiful*.

|  | ***Beautiful*** | ***Impressive*** | ***Endangered*** | ***Cute*** | ***Dangerous*** | ***Rare*** |
| --- | --- | --- | --- | --- | --- | --- |
| ***Beautiful*** | - | - | - | - | - | - |
| ***Impressive*** | 0.68 | - | - | - | - | - |
| ***Endangered*** | <2.2e-16 | <2.2e-16 | - | - | - | - |
| ***Cute*** | <2.2e-16 | <2.2e-16 | <2.2e-16 | - | - | - |
| ***Dangerous*** | <2.2e-16 | <2.2e-16 | <2.2e-16 | <2.2e-16 | - | - |
| ***Rare*** | <2.2e-16 | <2.2e-16 | <2.2e-16 | <2.2e-16 | <2.2e-16 | - |
