# Supplementary material for: The twenty most charismatic species
Source: PLoS One. 2018 Jul 9;13(7):e0199149. doi: 10.1371/journal.pone.0199149 (PMC6037359; doi:10.1371/journal.pone.0199149)
Supplement: S3 Table — Test of the difference between mean proportions of traits and proportions of species traits. See Fig 2. (DOCX) [file pone.0199149.s004.docx]

**S3 Table**: p-value of the Chi-square: test of the difference between mean proportions of traits and proportions of species traits. See Fig. 2.

| **Species** | **beau** | **impr** | **enda** | **cute** | **dang** | **rare** |  |  |
| --- | --- | --- | --- | --- | --- | --- | --- | --- |
| **tiger** | 0.3265 | 0.003891 | 0.798 | < 2.2e-16 | < 2.2e-16 | 5.352e-05 |  |  |
| **lion** | 2.421e-05 | < 2.2e-16 | < 2.2e-16 | < 2.2e-16 | < 2.2e-16 | < 2.2e-16 |  | Inf |
| **elephant** | 5.604e-11 | < 2.2e-16 | < 2.2e-16 | < 2.2e-16 | < 2.2e-16 | 5.267e-12 |  | Sup |
| **giraffe** | < 2.2e-16 | < 2.2e-16 | < 2.2e-16 | < 2.2e-16 | < 2.2e-16 | 5.863e-10 |  | Non sign |
| **leopard** | 8.936e-14 | 0.005543 | 1.489e-12 | < 2.2e-16 | < 2.2e-16 | 0.01159 |  |  |
| **panda** | < 2.2e-16 | < 2.2e-16 | < 2.2e-16 | < 2.2e-16 | < 2.2e-16 | < 2.2e-16 |  |  |
| **cheetah** | 2.066e-06 | 0.9993 | 1.048e-05 | 2.728e-08 | 4.819e-06 | 0.5824 |  |  |
| **polar bear** | 9.518e-05 | 0.0009435 | 2.532e-11 | 3.265e-09 | 0.01732 | 0.0004102 |  |  |
| **wolf** | 0.0003827 | 5.82e-07 | 6.79e-05 | < 2.2e-16 | 0.3478 | 6.427e-06 |  |  |
| **gorilla** | < 2.2e-16 | 1.799e-05 | < 2.2e-16 | < 2.2e-16 | 2.424e-05 | 6.498e-11 |  |  |
| **chimpanzee** | 5.092e-10 | 0.9062 | 0.02104 | < 2.2e-16 | 6.809e-12 | 0.04797 |  |  |
| **zebra** | < 2.2e-16 | < 2.2e-16 | 4.133e-08 | < 2.2e-16 | < 2.2e-16 | 0.1472 |  |  |
| **hippo** | 8.851e-10 | 2.293e-09 | 5.012e-06 | 4.09e-05 | 0.1307 | 0.61 |  |  |
| **shark** | 0.0002637 | 0.01367 | 0.812 | 1.163e-08 | 4.087e-12 | 0.7277 |  |  |
| **croco** | < 2.2e-16 | < 2.2e-16 | < 2.2e-16 | < 2.2e-16 | < 2.2e-16 | 1.699e-10 |  |  |
| **dolphin** | < 2.2e-16 | < 2.2e-16 | 0.1608 | < 2.2e-16 | < 2.2e-16 | < 2.2e-16 |  |  |
| **rhino** | < 2.2e-16 | 8.741e-06 | 3.94e-08 | < 2.2e-16 | 8.834e-05 | 0.0001604 |  |  |
| **bear** | 0.7747 | 0.6394 | 0.573 | 0.7452 | 0.1387 | 0.01329 |  |  |
| **koala** | < 2.2e-16 | < 2.2e-16 | 5.295e-07 | < 2.2e-16 | < 2.2e-16 | 4.869e-14 |  |  |
| **blue whale** | 0.5065 | 0.003043 | 0.006301 | 3.668e-08 | 2.717e-12 | 7.373e-05 |  |  |
