# Supplementary material for: The twenty most charismatic species
Source: PLoS One. 2018 Jul 9;13(7):e0199149. doi: 10.1371/journal.pone.0199149 (PMC6037359; doi:10.1371/journal.pone.0199149)
Supplement: S4 Table — Illustration in Fig 4. (DOCX) [file pone.0199149.s005.docx]

S4 Table: Estimates of the GLMM, illustrated in Fig. 4

|  | **Rare** | | | | **Dangerous** | | | | **Endangered** | | | | **Cute** | | | | **Impressive** | | | | **Beautiful** | | | |
| --- | --- | --- | --- | --- | --- | --- | --- | --- | --- | --- | --- | --- | --- | --- | --- | --- | --- | --- | --- | --- | --- | --- | --- | --- |
|  | Est. | Std, Error | z value | Pr(>\|z\|) | Est. | Std, Error | z value | Pr(>\|z\|) | Est. | Std, Error | z value | Pr(>\|z\|) | Est. | Std, Error | z value | Pr(>\|z\|) | Est. | Std, Error | z value | Pr(>\|z\|) | Estimate | Std, Error | z value | Pr(>\|z\|) |
| **BlueWhale(Intercept)** | **-0.197** | **0,406** | **-0,485** | **0,627** | **-3,716** | **0,506** | **-7,345** | **2,05E-13** | 0,859 | 0,244 | 3,515 | 4,39E-04 | -4,104 | 0,666 | -6,159 | 7,32E-10 | **0,179** | **1,500** | **0,119** | **0,905** | 0,610 | 0,218 | 2,798 | 5,14E-03 |
| **Brown Bear** | -1.530 | 0,257 | -5,954 | 2,62E-09 | 3,319 | 0,504 | 6,588 | 4,45E-11 | -0,978 | 0,248 | -3,942 | 8,08E-05 | 3,049 | 0,581 | 5,251 | 1,51E-07 | -1,747 | 0,314 | -5,568 | 2,57E-08 | -0,485 | 0,234 | -2,072 | 3,83E-02 |
| **Cheetah** | -1.024 | 0,195 | -5,249 | 1,53E-07 | 3,563 | 0,487 | 7,315 | 2,57E-13 | -1,421 | 0,214 | -6,628 | 3,39E-11 | 2,240 | 0,566 | 3,954 | 7,68E-05 | -1,661 | 0,283 | -5,864 | 4,52E-09 | 0,150 | 0,200 | 0,750 | 4,53E-01 |
| **Chimpanzee** | -1,667 | 0,229 | -7,293 | 3,04E-13 | 1,260 | 0,523 | 2,409 | 0,016 | -1,248 | 0,228 | -5,465 | 4,62E-08 | 4,436 | 0,566 | 7,843 | 4,39E-15 | -2,155 | 0,295 | -7,310 | 2,67E-13 | -1,972 | 0,225 | -8,768 | < 2e-16 |
| **Crocodile** | -2,340 | 0,233 | -10,055 | < 2e-16 | 5,557 | 0,497 | 11,173 | < 2e-16 | -2,569 | 0,231 | -11,114 | < 2e-16 | **-0,050** | **0,663** | **-0,075** | **0,940** | -1,058 | 0,291 | -3,638 | 2,75E-04 | -2,630 | 0,227 | -11,572 | < 2e-16 |
| **Dolphin** | -2,335 | 0,194 | -12,067 | < 2e-16 | -1,416 | 0,581 | -2,439 | 0,015 | -1,565 | 0,206 | -7,614 | 2,65E-14 | 4,530 | 0,557 | 8,133 | 4,18E-16 | -2,876 | 0,277 | -10,386 | < 2e-16 | **-0,069** | **0,189** | **-0,364** | **7,16E-01** |
| **Elephant** | -1,485 | 0,182 | -8,154 | 3,52E-16 | 2,283 | 0,484 | 4,720 | 2,36E-06 | -0,646 | 0,203 | -3,174 | 1,50E-03 | 1,954 | 0,559 | 3,494 | 4,76E-04 | **-0,388** | **0,277** | **-1,402** | **0,161** | -0,998 | 0,186 | -5,366 | 8,06E-08 |
| **Giraffe** | -1,979 | 0,193 | -10,268 | < 2e-16 | **-0,497** | **0,537** | **-0,926** | **0,355** | -2,570 | 0,211 | -12,170 | < 2e-16 | 3,240 | 0,558 | 5,802 | 6,53E-09 | -1,660 | 0,277 | -5,988 | 2,13E-09 | **-0,342** | **0,190** | **-1,803** | **0,071** |
| **Gorilla** | **-0,271** | **0,190** | **-1,43** | **0,153** | 2,871 | 0,487 | 5,890 | 3,86E-09 | **0,243** | **0,218** | **1,112** | **0,266** | 2,081 | 0,568 | 3,666 | 2,47E-04 | -0,807 | 0,285 | -2,832 | 4,62E-03 | -1,225 | 0,196 | -6,238 | 4,42E-10 |
| **Great White Shark** | -0,890 | 0,265 | -3,361 | 7,76E-04 | 5,024 | 0,530 | 9,486 | < 2e-16 | -0,727 | 0,274 | -2,654 | 7,95E-03 | -10,420 | 5,354 | -1,947 | 5,16E-02 | **0,007** | **0,403** | **0,019** | **0,985** | -1,066 | 0,261 | -4,083 | 4,45E-05 |
| **Hippo** | -1,311 | 0,216 | -6,077 | 1,23E-09 | 2,866 | 0,494 | 5,800 | 6,63E-09 | -2,236 | 0,234 | -9,542 | < 2e-16 | 3,144 | 0,569 | 5,530 | 3,20E-08 | -1,446 | 0,294 | -4,916 | 8,83E-07 | 1,954 | 0,222 | -8,819 | < 2e-16 |
| **Koala** | -0,475 | 0,194 | -2,454 | 0,014 | **-0,683** | **0,606** | **-1,128** | **0,259** | -0,795 | 0,215 | -3,696 | 2,19E-04 | 6,377 | 0,569 | 11,210 | < 2e-16 | -5,108 | 0,337 | -15,170 | < 2e-16 | -1,742 | 0,203 | -8,580 | < 2e-16 |
| **Leopard** | -0,661 | 0,183 | -3,61 | 3,06E-04 | 3,911 | 0,484 | 8,087 | 6,10E-16 | -1,292 | 0,206 | -6,276 | 3,48E-10 | 1,955 | 0,561 | 3,484 | 4,95E-04 | -1,641 | 0,277 | -5,929 | 3,04E-09 | 0,482 | 0,191 | 2,516 | 0,012 |
| **Lion** | -1,838 | 0,184 | -9,988 | < 2e-16 | 4,320 | 0,483 | 8,949 | < 2e-16 | -1,980 | 0,204 | -9,698 | < 2e-16 | 1,566 | 0,560 | 2,796 | 5,18E-03 | -0,933 | 0,276 | -3,384 | 7,15E-04 | **-0,250** | **0,186** | **-1,341** | **0,180** |
| **Panda Bear** | **0,307** | **0,184** | **1,668** | **0,095** | 1,124 | 0,494 | 2,277 | 0,023 | **0,354** | **0,211** | **1,677** | **0,093** | 5,155 | 0,558 | 9,237 | < 2e-16 | -3,634 | 0,282 | -12,891 | < 2e-16 | -1,005 | 0,190 | -5,288 | 1,23E-07 |
| **Polar Bear** | **-0,259** | **0,189** | **-1,372** | **0,169** | 3,797 | 0,486 | 7,814 | 5,53E-15 | 0,665 | 0,223 | 2,988 | 2,81E-03 | 2,780 | 0,562 | 4,950 | 7,42E-07 | -1,318 | 0,282 | -4,680 | 2,86E-06 | **-0,186** | **0,196** | **-0,950** | **3,42E-01** |
| **Rhino** | -0,606 | 0,201 | -3,017 | 2,55E-03 | 3,520 | 0,490 | 7,181 | 6,92E-13 | **-0,385** | **0,223** | **-1,726** | **0,084** | **1,034** | **0,605** | **1,709** | **0,087** | -1,022 | 0,293 | -3,490 | 4,83E-04 | -1,983 | 0,215 | -9,238 | < 2e-16 |
| **Tiger** | -0,393 | 0,180 | -2,186 | 0,029 | 4,227 | 0,483 | 8,759 | < 2e-16 | -0,443 | 0,204 | -2,176 | 0,029 | 1,912 | 0,559 | 3,420 | 6,26E-04 | -1,158 | 0,275 | -4,209 | 2,57E-05 | **0,299** | **0,187** | **1,599** | **0,11** |
| **Wolf** | -0,756 | 0,183 | -4,140 | 3,48E-05 | 3,143 | 0,484 | 6,499 | 8,07E-11 | -0,820 | 0,205 | -3,993 | 6,51E-05 | 2,072 | 0,560 | 3,699 | 2,17E-04 | -2,093 | 0,276 | -7,581 | 3,43E-14 | **-0,275** | **0,188** | **-1,462** | **0,144** |
| **Zebra** | -1,929 | 0,223 | -8,640 | < 2e-16 | **-1,198** | **0,738** | **-1,624** | **0,104** | -2,708 | 0,238 | -11,386 | < 2e-16 | 3,884 | 0,563 | 6,895 | 5,38E-12 | -4,080 | 0,312 | -13,056 | < 2e-16 | -0,408 | 0,206 | -1,984 | 0,047 |
