# Supplementary material for: The twenty most charismatic species
Source: PLoS One. 2018 Jul 9;13(7):e0199149. doi: 10.1371/journal.pone.0199149 (PMC6037359; doi:10.1371/journal.pone.0199149)
Supplement: S6 Table — (DOCX) [file pone.0199149.s007.docx]

**S6 Table:** MCA2 (Species traits VS respondents traits) :

| **Eigenvalues** | **Dim.1** | **Dim.2** | **Dim.3** | **Dim.4** | **Dim.5** | **Dim.6** | **Dim.7** | **Dim.8** | **Dim.9** | **Dim.10** |
| --- | --- | --- | --- | --- | --- | --- | --- | --- | --- | --- |
| **Variance** | 0.417 | 0.340 | 0.284 | 0.262 | 0.255 | 0.248 | 0.229 | 0.209 | 0.169 | 0.089 |
| **% of Variance** | 16.686 | 13.586 | 11.349 | 10.490 | 10.190 | 9.912 | 9.155 | 8.340 | .6751 | 3.541 |
| **Cumulative % of variance** | 16.686 | 30.272 | 41.622 | 52.112 | 62.301 | 72.213 | 81.368 | 89.708 | 96.459 | 100 |
